# Corticosteroid Use and Complications in a US Inflammatory Bowel Disease Cohort

**DOI:** 10.1371/journal.pone.0158017

**Published:** 2016-06-23

**Authors:** Akbar K. Waljee, Wyndy L. Wiitala, Shail Govani, Ryan Stidham, Sameer Saini, Jason Hou, Linda A. Feagins, Nabeel Khan, Chester B. Good, Sandeep Vijan, Peter D. R. Higgins

**Affiliations:** 1 VA Center for Clinical Management Research, VA Ann Arbor Health Care System, Ann Arbor, MI, United States of America; 2 Department of Internal Medicine, Division of Gastroenterology and Hepatology, University of Michigan Health System, Ann Arbor, MI, United States of America; 3 Department of Internal Medicine, Division of Gastroenterology and Hepatology, Houston VA HSR&D Center of Excellence, Michael E. DeBakey Veterans Affairs Medical Center, Houston, TX, United States of America; 4 Department of Medicine, Baylor College of Medicine Medical Center, Houston, TX, United States of America; 5 Department of Internal Medicine, Division of Gastroenterology and Hepatology, VA North Texas Health Care System, Dallas, TX, United States of America; 6 Divisions of Gastroenterology and Hepatology, University of Texas Southwestern Medical Center at Dallas, Dallas, TX, United States of America; 7 Department of Internal Medicine, Division of Gastroenterology, Philadelphia VA Medical Center, Philadelphia, PA, United States of America; 8 Department of Medicine, University of Pennsylvania Perelman School of Medicine, Philadelphia, PA, United States of America; 9 Center for Health Equity Research and Promotion, Veterans Affairs Pittsburgh Healthcare System, Pittsburgh, PA, United States of America; 10 Department of Medicine, University of Pittsburgh School of Medicine, Pittsburgh, PA, United States of America; CWRU/UH Digestive Health Institute, UNITED STATES

## Abstract

**Background and Aims:**

Corticosteroids are effective for the short-term treatment of inflammatory bowel disease (IBD). Long-term use, however, is associated with significant adverse effects. To define the: (1) frequency and duration of corticosteroid use, (2) frequency of escalation to corticosteroid-sparing therapy, (3) rate of complications related to corticosteroid use, (4) rate of appropriate bone density measurements (dual energy X-ray absorptiometry [DEXA] scans), and (5) factors associated with escalation and DEXA scans.

**Methods:**

Retrospective review of Veterans Health Administration (VHA) data from 2002–2010.

**Results:**

Of the 30,456 Veterans with IBD, 32% required at least one course of corticosteroids during the study time period, and 17% of the steroid users had a prolonged course. Among these patients, only 26.2% underwent escalation of therapy. Patients visiting a gastroenterology (GI) physician were significantly more likely to receive corticosteroid-sparing medications. Factors associated with corticosteroid-sparing medications included younger age (OR = 0.96 per year,95%CI:0.95, 0.97), male gender (OR = 2.00,95%CI:1.16,3.46), GI visit during the corticosteroid evaluation period (OR = 8.01,95%CI:5.85,10.95) and the use of continuous corticosteroids vs. intermittent corticosteroids (OR = 2.28,95%CI:1.33,3.90). Rates of complications per 1000 person-years after IBD diagnosis were higher among corticosteroid users (venous thromboembolism [VTE] 9.0%; fragility fracture 2.6%; Infections 54.3) than non-corticosteroid users (VTE 4.9%; fragility fracture 1.9%; Infections 26.9). DEXA scan utilization rates among corticosteroid users were only 7.8%.

**Conclusions:**

Prolonged corticosteroid therapy for the treatment of IBD is common and is associated with significant harm to patients. Patients with prolonged use of corticosteroids for IBD should be referred to gastroenterology early and universal efforts to improve the delivery of high quality care should be undertaken.

## Introduction

Corticosteroids are powerful, non-selective systemic anti-inflammatory drugs that are frequently used to treat autoimmune conditions. In inflammatory bowel disease (IBD), corticosteroids are the mainstay of “rescue” therapy for patients who are experiencing a disease flare. Despite the efficacy of these medications in the short-term, corticosteroids are known to cause serious adverse effects with long term use including bone loss [1], venous thromboembolism (VTE) [2] and poor wound healing [3]. Corticosteroids often lead to rapid resolution of IBD symptoms, which aligns with patient preferences for treatment. However, this may lead to inappropriate use of these medications for maintenance when other treatment options may be better in the longer-term. Escalation of therapy to corticosteroid-sparing maintenance therapy such as immunomodulators [4, 5] or biological agents [6, 7] can improve disease outcomes and avoid the complications of prolonged steroid use. Underutilization of corticosteroid-sparing medications and failure to monitor for complications of corticosteroid use have motivated professional subspecialty societies to advocate for reduction in corticosteroid use and early initiation of corticosteroid-sparing therapy [8]. Indeed, early use of corticosteroid-sparing medications is now viewed as an increasingly important measure of the quality of IBD care [9]. These measures of the quality of the process of IBD care are aimed at optimizing medication use and improving patient safety by minimizing exposure to medications associated with both short and long term complications [10]. According to Plevy *et al*. patients with two corticosteroid courses within one year should escalate to corticosteroid-sparing maintenance therapy [11]. The American Gastroenterological Association (AGA) recommends dual X-ray absorptiometry (DEXA) scan evaluation of bone density in any patient on corticosteroids for at least 3 months or recurrent courses, any male with IBD over the age of 50 or any post-menopausal female. [12, 13].

However, little is known about the frequency of corticosteroid use, the frequency of escalation to corticosteroid-sparing therapy among corticosteroid users and the rate of complications associated with corticosteroid use. The Veterans Health Administration (VHA) provides a unique opportunity to evaluate and study corticosteroid use due to the large number of Veterans with IBD in this population and the availability of detailed electronic medical record data, including medication use. Understanding the link between processes of care (corticosteroid-sparing maintenance use) and outcomes (rate of corticosteroid complications) is particularly important in an era where Accountable Care Organizations (ACOs) will emphasize the delivery of high quality care and where there may be an increasing push to tie process measures to reimbursement. The aims of this study were to quantify: (1) frequency and duration of corticosteroid use in a national cohort of IBD patients in the VHA; (2) the frequency of appropriate escalation to corticosteroid-sparing maintenance therapy; (3) the rate of complications related to corticosteroid use, (4) the rate of appropriate bone density measurements (DEXA scans), and (5) factors associated with escalation and DEXA scans.

## Methods

### Overview

We used an Institutional Review Board (IRB) approved, national Veterans Health Administrative electronic data to conduct a retrospective cohort study of patients with inflammatory bowel diseases. Patients were identified using previously validated algorithms based on a combination of inpatient and outpatient International Classification of Diseases, Ninth Revision, Clinical Modification (ICD-9-CM) codes for Crohn’s disease (CD 555.x), and ulcerative colitis (UC 556.x) [14]. Patients were selected for inclusion if they had two or more of these ICD-9-CM codes during at least two clinical encounters between 2002–2009, with at least one of these encounters being an outpatient visit. This approach has a positive predictive value for Crohn’s disease of 0.84 and a positive predictive value for UC of 0.91 in the VHA [14]. Veterans were classified as CD if all ICD-9-CM codes were 555.x and UC if all codes were 556.x and the remaining Indeterminate colitis (IC). The date of the first encounter with an IBD ICD-9-CM code was considered the Veterans “IBD index date” for study purposes. Patients were identified with an IBD diagnosis between 2002 and 2009 and monitored for corticosteroid use during 2002–2010. We also extracted data on medication use, imaging, and visits for gastroenterology (GI) specialty care.

### Data On Medication Use and Gastroenterology Specialty Care Visits

We extracted data on filled prescriptions for both oral and intravenous corticosteroids of various generic names (Appendix A in S1 Appendices) from the VHA Decision Support Systems (DSS) National Data Extraction data source. We also determined the indication for corticosteroids by searching for ICD-9-CM codes for a variety of common inflammatory comorbid conditions in the 7 days prior to the prescription fill date (Appendix B in S1 Appendices). In addition, we excluded corticosteroid dose packs and fills that were short-term or low dose (fills of 10 mg for two weeks or less, and fills where the dose was ≤2 mg for more than one week). In order to ensure the accuracy of this administrative dataset, manual chart review was performed to validate each corticosteroid prescription for IBD within 3 selected VHA facilities in Veterans Integrated Service Network (VISN) 11 (Ann Arbor, MI; Battle Creek, MI; and Saginaw, MI) in addition to an ~10% random sample of other VHA facilities.

### Outcome Variable Definitions

#### Corticosteroid User Groups

The primary outcome of interest was the use of corticosteroids in Veterans with IBD over the study period. Additional analyses were also performed to describe the patterns of corticosteroids use and are presented in the Supplementary Results “Patterns of Corticosteroid Use” in S1 Tables. Corticosteroid users were classified into continuous corticosteroid users (CS), intermittent corticosteroid users (IS), or any corticosteroid users (AS), according to the specifications outlined below. We also examined the use of corticosteroids for other conditions (OS).

**Fig 1:**
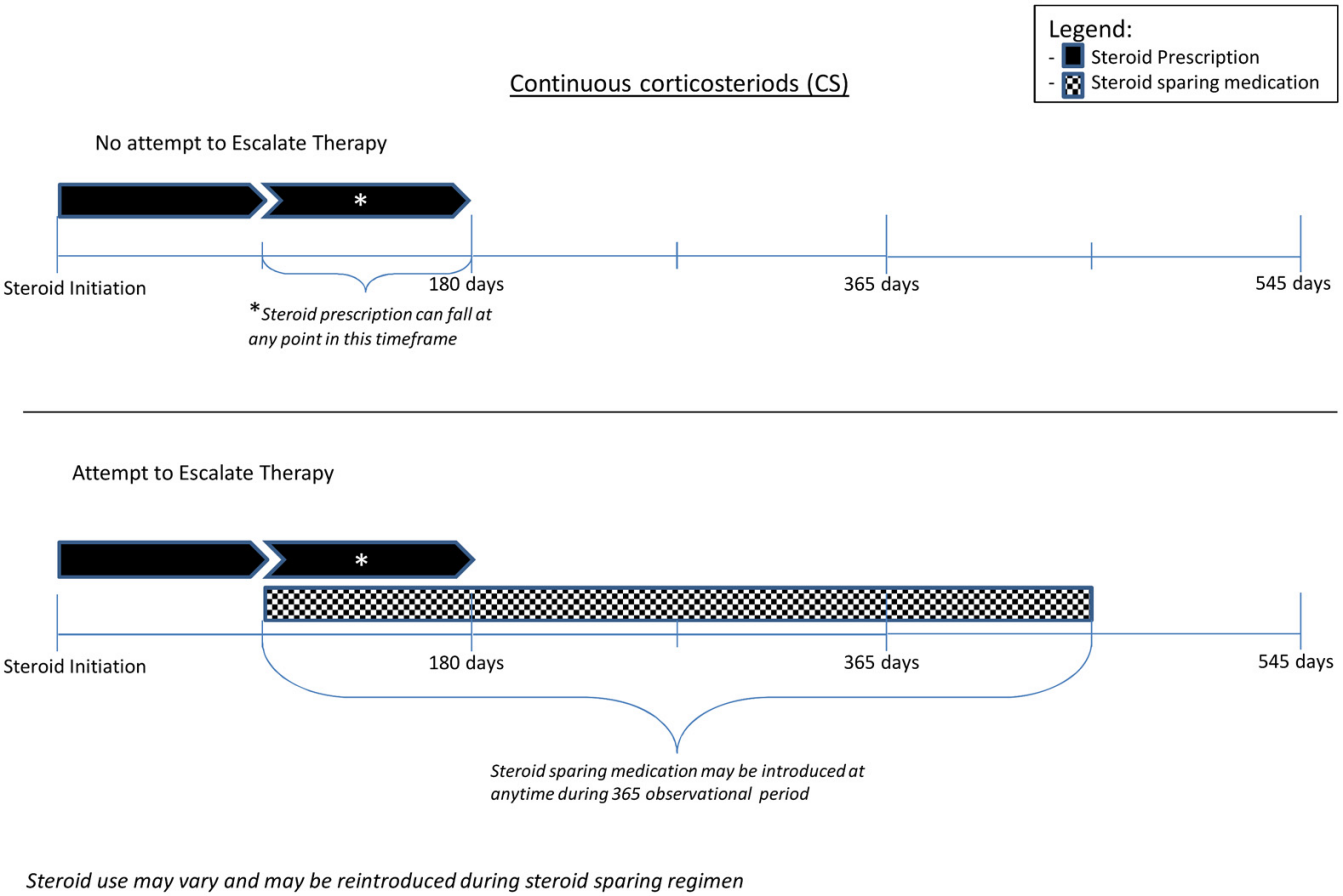
Escalation to corticosteroid-sparing therapy for Veterans who were continuous corticosteroid users (CS).

**Fig 2:**
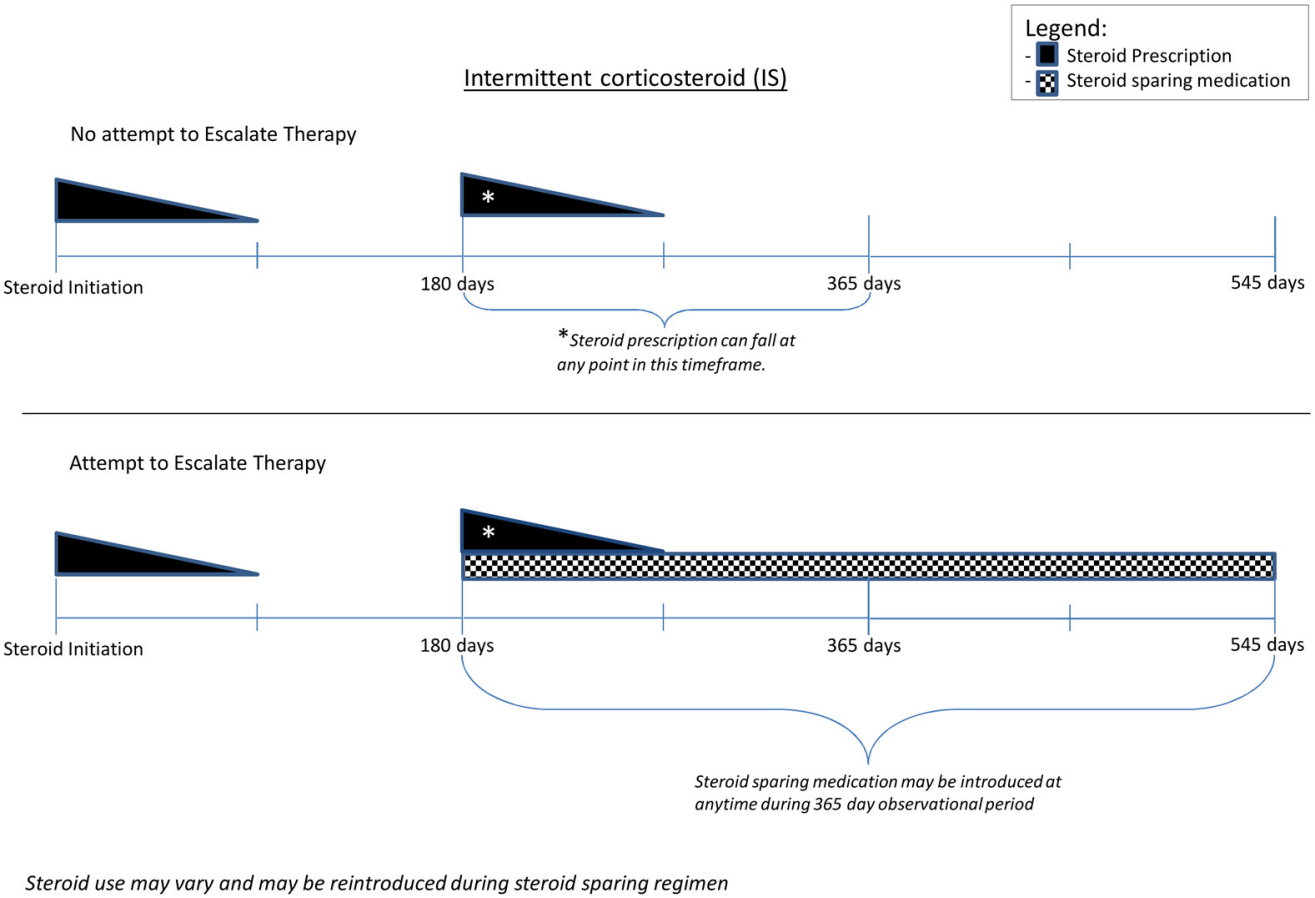
Escalation to corticosteroid-sparing therapy for Veterans who were intermittent corticosteroid users (IS).

We used the date of first prescription of corticosteroids within our study period as the index date. CS was defined as patients who were initiated on corticosteroids and treated for up to 90 days (at least 10 mg of corticosteroids for 2 weeks) and subsequently required corticosteroids again within 90-days. IS was defined as those who were initiated on corticosteroids and treated for up to 90 days, then had at least 90 days without steroids, but subsequently required additional steroids within the 365-day evaluation period after initiation of corticosteroids. Figs 1 and 2 illustrate the definitions of CS and IS, respectively. We excluded patients from the CS or IS if they were prescribed any corticosteroid-sparing medication during the 365 days prior to the initiation of second dose of corticosteroids and defined these patients as AS. OS comprised of patients who required at least one corticosteroid prescription for a non-IBD condition.

#### Rate of Escalation to Corticosteroid-sparing Therapy for IBD

Our primary analysis involved examining the rate of escalation of therapy among Veterans that were prescribed corticosteroids for their IBD (CS, IS, AS). Escalation of therapy was defined as the introduction of a corticosteroid-sparing medication (immunosuppressant or anti-tumor necrosis factor [anti-TNF], or a combination of an anti-TNF and an immunomodulators if they were started within 90-days of each other) during the evaluation period of 365-days after the corticosteroid fill. See Figs 1 and 2 for an illustration of the therapeutic escalation for CS and IS.

#### Corticosteroid-Associated Complications in Veterans with IBD

In order to compare complication rates related to corticosteroids and IBD, we examined rates of venous thromboembolism (VTE), fragility fractures and infections among veterans who never received corticosteroids and patients who received any corticosteroids for their IBD (CS, IS, AS). The ICD-9-CM codes used to identify VTE, fragility fractures and infections are included in Appendix C in S1 Appendices. Complication rates were examined during three periods: 1) the year prior to the study period index IBD diagnosis, 2) the year after study period index IBD diagnosis (or to the corticosteroid initiation date in the event that corticosteroids were initiated within one year after IBD diagnosis) and 3) year after initiation of corticosteroids for the corticosteroid group.

#### Healthcare Maintenance: DEXA Scan Use in Veterans with IBD on Corticosteroids

To examine healthcare maintenance we identified DEXA scans performed within 1 year after the use of corticosteroids using Current Procedural Terminology (CPT)codes (pre-2006) 76075 and (post-2006) 77080. In order to compare utilization of corticosteroid-related healthcare maintenance, we determined the frequency of DEXA scans among all patients (CS, IS, AS and OS) who were on corticosteroids and those who were not. We further subdivided the analysis by age less than and greater than 50 as the AGA guidelines recommend DEXA scans for all IBD patients above the age of 50 regardless of corticosteroid utilization.

#### Ethics Statement

The work described was approved by the Research and Development committee at VA Ann Arbor Health Care System. A waiver of authorization was approved for a retrospective review of existing medical record data. All data was de-identified prior to analysis.

## Statistical Analysis

Descriptive statistics were used to compare patient characteristics among the corticosteroid and non-corticosteroid groups comparing the patient age and whether or not a visit with a GI specialist occurred. Demographic and clinical variables were compared between the corticosteroid groups and additional supplementary analysis was done among the various patterns of corticosteroid users (CS, IS, AS) and NS (no corticosteroid users). Independent sample t-tests were used to compare corticosteroid user groups on continuous measures. Negative binomial regression models were used to estimate the effects of corticosteroid users on count outcomes. Pearson’s chi-square tests were used to test the relationship between corticosteroid users and categorical measures. In the event that cell sizes dropped below n = 5, Fisher’s exact test was used to test for statistical significance. We used Poisson regression models to assess the impact of IBD corticosteroid use on complications, adjusting for age, male gender, Charlson comorbidity index, and exposure time (IBD diagnosis date or corticosteroid initiation date to the end of the study period). To assess escalation of therapy and DEXA scan use within one year of corticosteroid initiation, we used logistic regression to evaluate Veteran predictors (age, gender and GI visit pre-corticosteroid initiation) of outcomes and adjusting for clustering by facility. In addition, we conducted a multilevel logistic regression model, adjusting for Veteran level covariates (age, gender), to assess the facility-level variation in escalation of therapy for Veterans among all corticosteroids users (CS, IS, AS). All data analysis was performed using Stata 13.1 (StataCorp, College Station, TX).

## Results

We identified 30,456 patients with IBD over the study period. The mean (±SD) age was 60 (15) years. The majority of Veterans were male (94%) and Caucasian (69%). Additional baseline characteristics are provided in Table 1 and Supplementary Table A in S1 Tables for the various patterns of corticosteroid users.

**Table 1:** Patient Characteristics of no corticosteroid and all corticosteroid users among IBD Veterans.

	Total	No Corticosteroid Users (NS)	All Corticosteroid Users *	P-value
No. of Patients, N (%)	30,456 (100)	20,575 (67.6)	9,881 (32.4)	
Ulcerative Colitis, N (%)	10,664 (35.0)	7,126 (34.6)	3,538 (35.8)	0.045
Crohn’s Disease, N (%)	16,429 (53.9)	11,948 (58.1)	4,481 (45.3)	<0.001
Indeterminate Colitis, N (%)	3,363 (11.0)	1,501 (7.3)	1,862 (18.8)	<0.001
Male, N (%)	28,500 (93.6)	19,389 (94.2)	9,111 (92.2)	<0.001
Age, years Mean (SD)	60.3 (15.3)	62.3 (14.5)	56.2 (16.0)	<0.001
Race, N (%)				<0.001
Caucasian	21,010 (69.0)	13,898 (67.5)	7,112 (72.0)	
African American	2,097 (6.9)	1,199 (5.8)	898 (9.1)	
Other	491 (1.6)	298 (1.4)	193 (2.0)	
Unknown or Missing	6,858 (22.5)	5,180 (25.2)	1,678 (17.0)	
Any GI Visit, N (%)	15,832 (51.9)	8,332 (40.5)	7,500 (75.9)	<0.001

* Includes CS, IS, AS, OS

### Rates of Corticosteroid Use

Among the 30,456 Veterans, 20,575 patients (68%) did not require any corticosteroids (NS group) during the study period for their inflammatory bowel disease or for any other coexisting condition (see Appendix B in S1 Appendices for coexisting conditions). The remaining 32% (n = 9,881) of Veterans received at least one fill of corticosteroids during the study period with various patterns of corticosteroid use (see Supplementary Results “Patterns of Corticosteroid Use” in S1 Tables). The manual chart review confirmed that ~90% of corticosteroid use was for inflammatory bowel disease and not for other coexisting conditions. In general, the patients who received steroids were younger than those who did not (56.2 years +/- 16 versus 62.0 years +/- 15, p<0.001). Corticosteroid users were more likely to have a GI visit during the study period (77.8%) compared to the NS group (43.2%), p<0.001.

### Rate of Escalation to Corticosteroid-sparing Therapy for IBD

**Fig 3:**
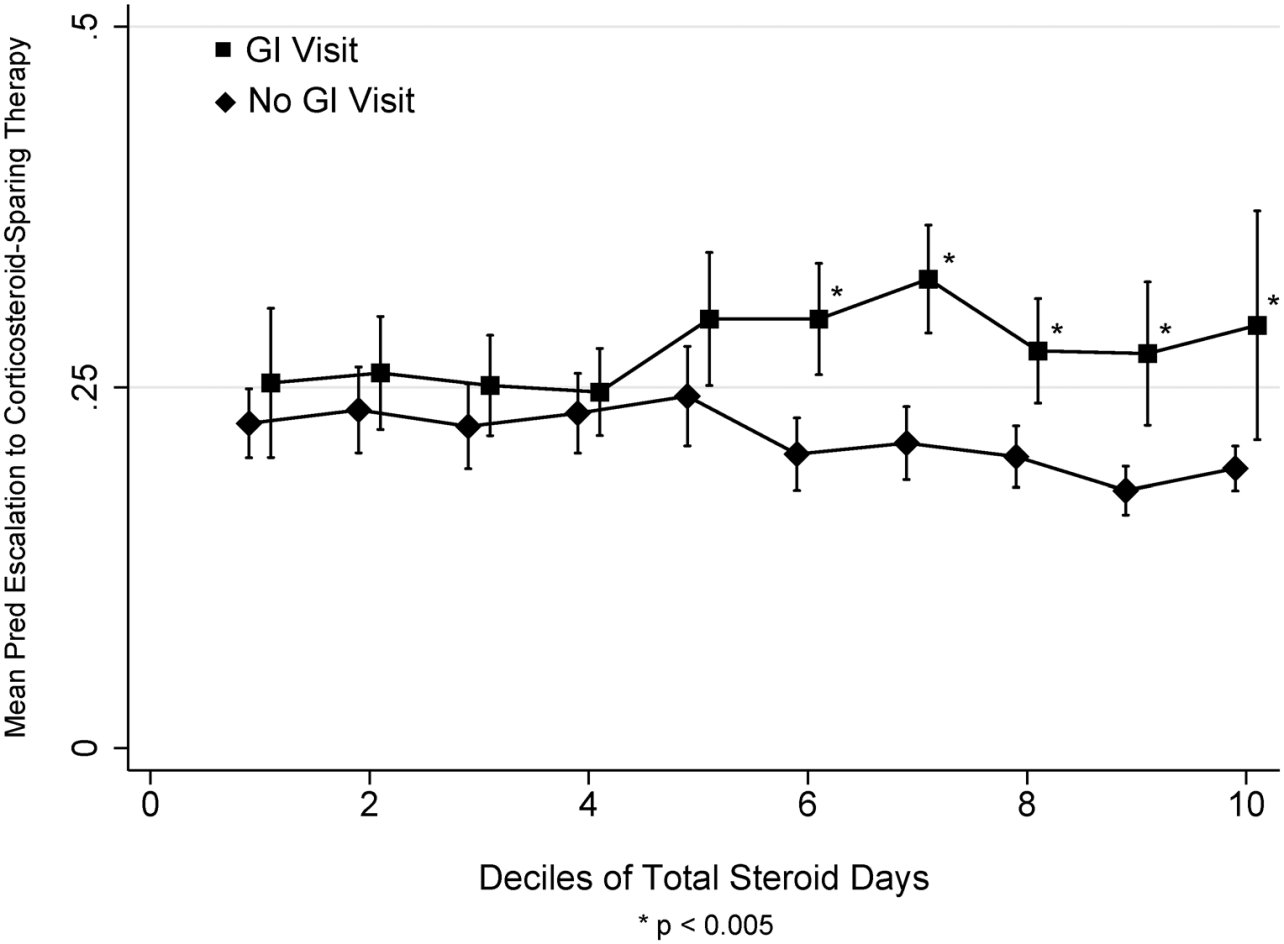
Mean predicted likelihood of escalation to corticosteroid-sparing therapy by deciles of Total Steroid Days among Veterans with a GI visit (square) vs. no GI visit (diamond) during the study period. Vertical lines (slightly offset for better visibility) represent 95% Confidence Intervals. Predicted escalation adjusted for age, male gender, the use of CS vs. IS, and clustering by facility.

The overall rate of escalation to corticosteroid sparing medication for IBD with immunosuppressants or anti-TNF therapy or the combination was lower among the NS group (5.9%) compared to the patients who received corticosteroids for their IBD (41.8%, p<0.001) (Table 2). Escalation rates were lower among CS and IS Veterans, where only 26.2% (439/1,678) of the patients were escalated to corticosteroid-sparing therapy in the year after their second corticosteroid prescription compared to the AS Veterans 47.7% (2,089/4,376) (Supplementary Table B in S1 Tables.) Veterans with CS and IS were much more likely to step up to corticosteroid-sparing therapy (immunomodulator or anti-TNF) if they had a GI visit vs. no GI visit during their periods of corticosteroid use (unadjusted rates 69.3% vs. 30.7% in CS group; 67.3% vs. 32.7 in IS group), p<0.001 (Table 3). Adjusting for age, male gender, steroid use group, and clustering by facility there was an increased likelihood of corticosteroid-sparing therapy in patients with a GI visit for those patients with a higher proportion of corticosteroid use (Fig 3).

**Table 2:** Corticosteroid user characteristics among Veterans with and without corticosteroids for IBD only.

	Total	No Corticosteroid Users (NS)	IBD Corticosteroid Users*	P-value
No. of Patients, N (%)	26,629 (100)	20,575 (77.3)	6,054 (22.7)	
Study Days, median (5–95 percentile)	2,048 (518–3,174)	2,032 (508–3,173)	2,097 (540–3,182)	<0.001
Rate of Escalation to corticosteroid-sparing therapy, N (%)	3,738 (14.0)	1,210 (5.9)	2,528 (41.8)	<0.001
Type of Escalation Medication (N = 3,738)				<0.001
Anti-TNF only	631 (2.4)	85 (7.0)	546 (21.6)	
Immunomodulator only	2,727 (10.2)	1,064 (87.9)	1,663 (65.8)	
Combination	380 (1.4)	61 (5.0)	319 (12.6)	
Charlson Comorbidity, Median(5–95 percentile)	0 (0–2)	0 (0–2)	0 (0–2)	

*Includes CS, IS, and AS

**Table 3:** Appropriate escalation to corticosteroid-sparing therapy vs. GI visit during the during corticosteroid evaluation period.

	GI Visit		
	No	Yes	P-value
Continuous Corticosteroid Use (N = 1,438)			<0.001
Not Escalated	877 (83.2)	177 (16.8)	
Escalated	118 (30.7)	266 (69.3)	
*Total*	*995 (69*.*2)*	*443 (30*.*8)*	
Intermittent Corticosteroid Use (N = 240)			<0.001
Not Escalated	149 (80.5)	36 (19.5)	
Escalated	18 (32.7)	37 (67.3)	
*Total*	*167 (69*.*6)*	*73 (30*.*4)*	

### Facility Level Variation

To examine the potential facility-level variation we conducted a multi-level logistic regression analysis using the IBD corticosteroid groups only without any prior corticosteroid-sparing therapy (e.g., CS, IS, AS) with the outcome of patient-level escalation (N = 5,789). Patients were clustered within 128 VA facilities, with an average of 45 corticosteroid patients per facility (SD = 29, min = 5, max = 133). For the purpose of these exploratory models, escalation was defined as the introduction of any corticosteroid-sparing medication during the year after the corticosteroid initiation date. We excluded patients who had a corticosteroid-sparing medication fill prior to the corticosteroid initiation date. The overall escalation rate was 41.2% across the three patterns of corticosteroid use. We first ran the empty model with a random-intercept for the facility to assess the unadjusted facility-level variation. We ran a second model adjusting for Veteran level covariates measured prior to corticosteroid initiation (age at IBD diagnosis, gender). The Intraclass Correlation (ICC) for the unadjusted model was 0.025 (95% CI: 0.014, 0.043) and for the adjusted model was 0.022 (95% CI: 0.012, 0.040). Thus, after adjusting for differences in patient-level covariates, 2.2% of the total variation in escalation rates was due to facility-level factors. The median odds ratio (MOR) was 1.30. This suggests that, given two randomly selected facilities, the Veteran at a facility with a higher escalation probability has a 30% greater odds of escalation then a similar matched (for age and gender) Veteran at a facility with a lower escalation probability.

### Corticosteroid-Associated Complications in Veterans with IBD

**Fig 4:**
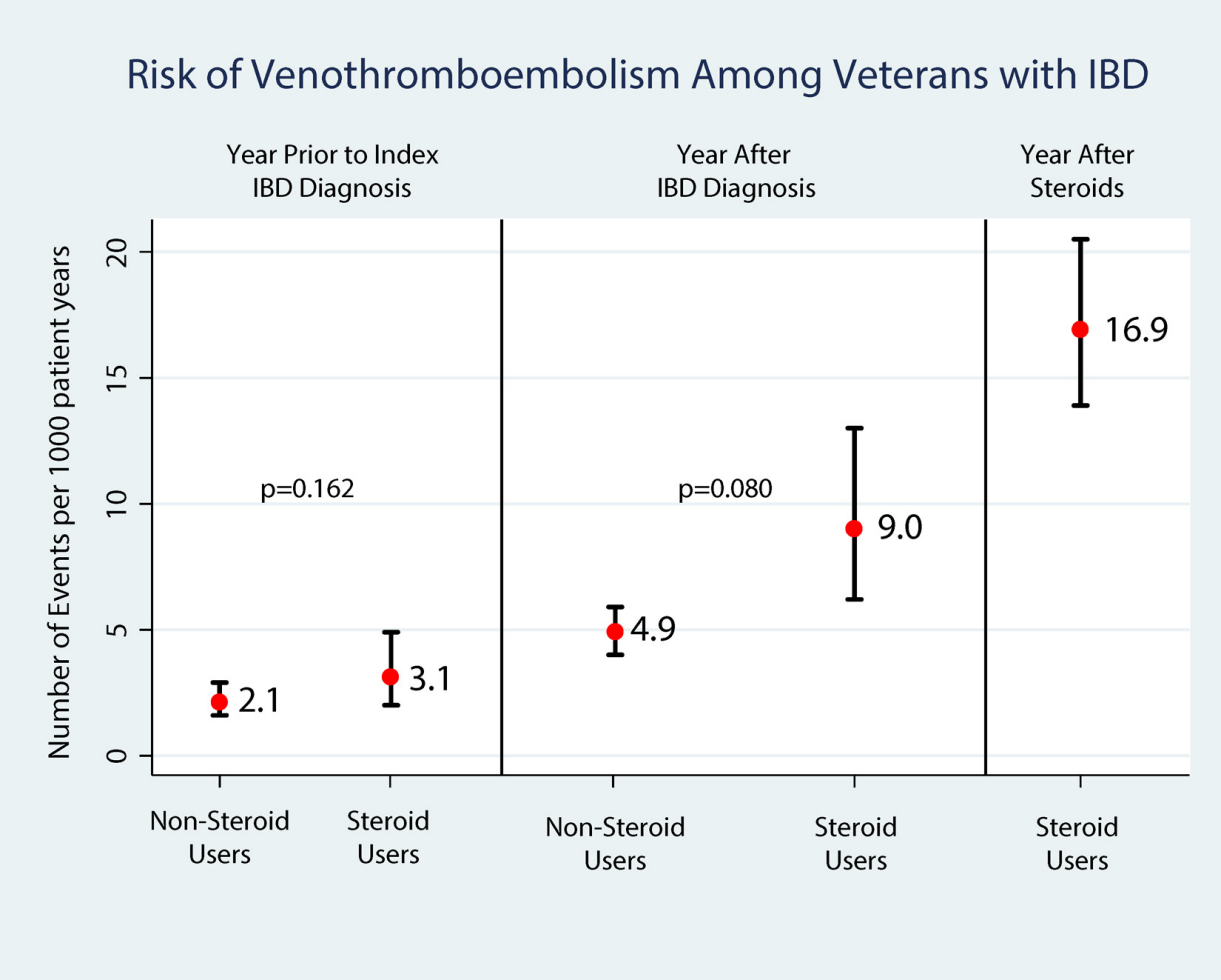
Risk of Venothromboembolism among Veterans with IBD.

**Fig 5:**
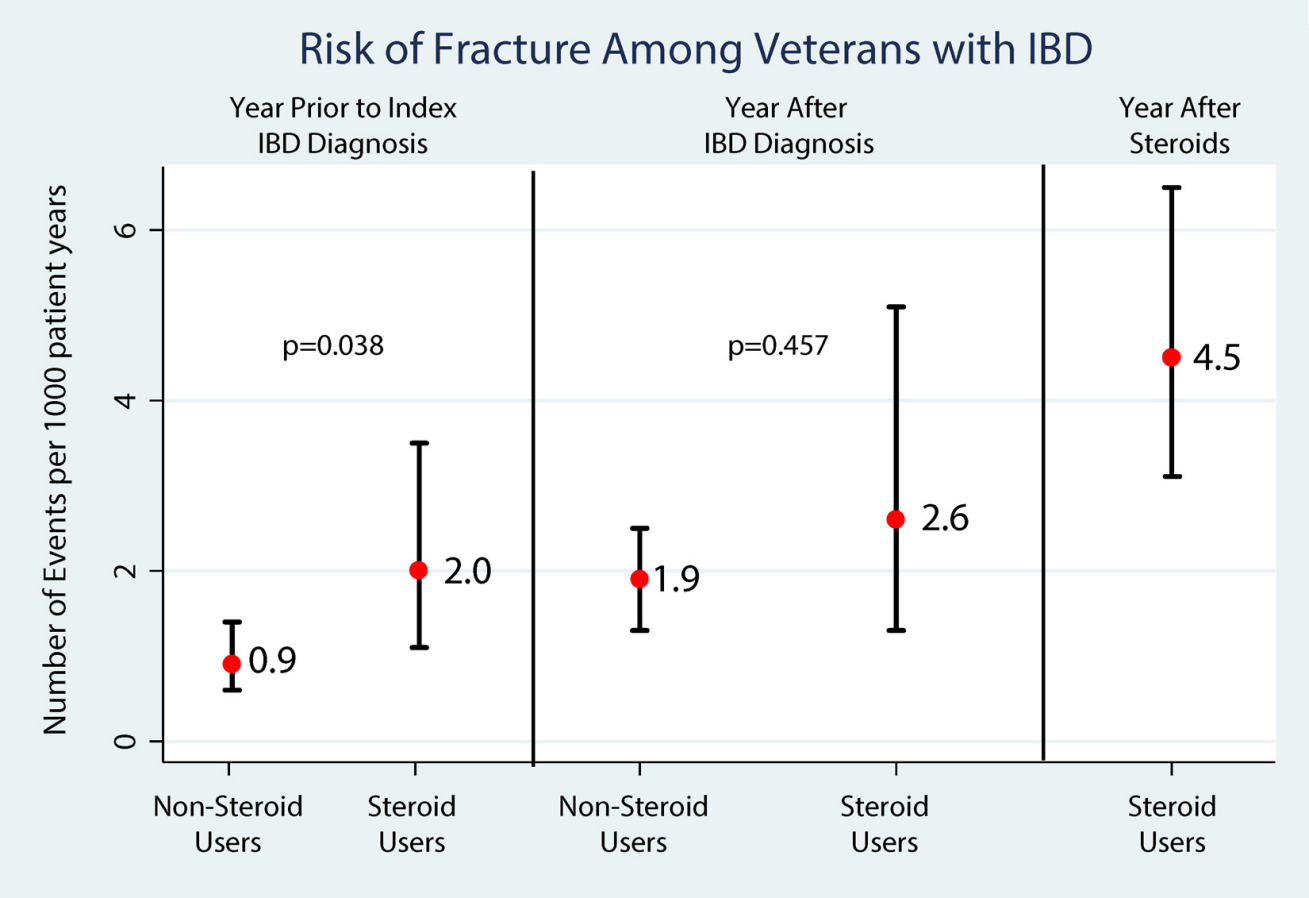
Risk of Fragility fractures among Veterans with IBD.

**Fig 6:**
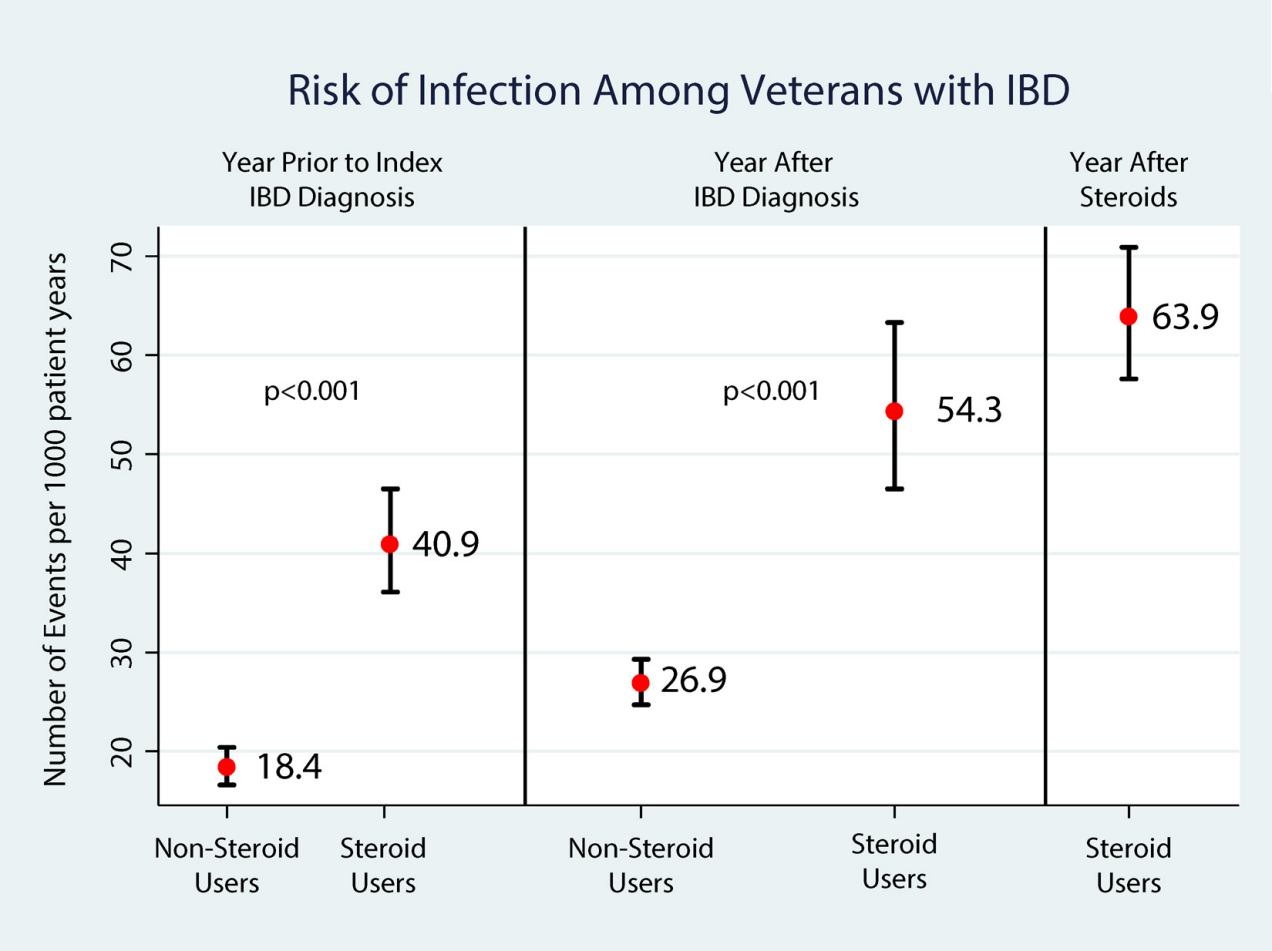
Risk of Infections among Veterans with IBD.

A descriptive comparison was used to evaluate the risk of VTE (Deep vein thrombosis [DVT]/Pulmonary embolism [PE]), fragility fractures and infections between those exposed to corticosteroids for their IBD (CS, IS, AS) and those with no corticosteroid exposure. Comparisons of the unadjusted rates for each complication in the year prior to diagnosis of IBD, in the year after their study period index diagnosis of IBD and in the year after the initiation of corticosteroids are shown in Figs 4, 5 and 6, respectively. VTE rates more than doubled (2.1 to 4.9 per 1000 person-years) after diagnosis among patients not exposed to corticosteroids. Among those with corticosteroid exposure, the rate of VTE almost tripled (3.1 to 9.0 per 1000 person-years) after study period index diagnosis and increased more than 5 fold (16.9 per 1000 person-years) in the year after corticosteroid exposure compared to the year prior to diagnosis. Similarly, fragility fracture rates doubled (0.9 to 1.9 per 1000 person-years) after study period index diagnosis among patients not exposed to corticosteroids. Among those with corticosteroid exposure, the rate of fragility fractures increased from 2.0 to 4.5 per 1000 person-years in the year after corticosteroid exposure compared to the year prior to diagnosis. Among those with corticosteroid exposure, the rate of infections increased from 40.9 to 63.9 per 1000 person-years in the year after corticosteroid exposure compared to the year prior to diagnosis. Adjusting for age, male gender, Charlson comorbidity index, we found that compared to the year after study period index IBD diagnosis, IBD corticosteroid users (N = 6,045) had 4.68 greater probability of experiencing a VTE (IRR = 4.68, 95%CI: 3.52, 6.24, p<0.001). Similarly, IBD corticosteroid users had a 3.7 greater probability of experiencing a fragility fracture (IRR = 3.72, 95% CI: 2.24, 6.19, p<0.001). In addition, IBD corticosteroid users had 2.85 greater probability of infection (IRR = 2.85, 95% CI: 2.48, 3.28, p<0.001). There was no difference between the rates of complications between the CS, IS and AS groups compared to the OS groups. (IRR = 0.83, 95%CI:0.61, 1.14, p = 0.256 for VTE, IRR = 0.83, 95%CI: 0.47, 1.47, p = 0.533 for fragility fractures and IRR = 0.92, 95%CI: 0.77, 1.00, p = 0.318 for Infections).

### Healthcare Maintenance: DEXA Scan Use in Veterans with IBD on Corticosteroids

As shown in Table 4, the rates of DEXA scanning were very low in the corticosteroid cohort; only 7.8% of these patients received a DEXA scan within one year of corticosteroid initiation, using the above definitions. Rates were similar between age groups; 8.0% (n = 546) of patients over 50 years old and 7.4% (n = 228) of patients 50 years and younger received a DEXA scan. Among those that were on corticosteroids but did not have a DEXA scan (N = 9,107), only 8.2% of Veterans had a prior bisphosphonate prescription fill.

**Table 4:** DEXA use among patients using corticosteroids (N = 9,881).

	DEXA SCAN 1 year after corticosteroid Initiation		
	No	Yes	p
Age			0.34
≤ 50 Yrs of Age	2,833 (92.6)	228 (7.4)	
> 50 Yrs of Age	6,274 (92.0)	546 (8.0)	
*Total*	9,107 (92.2)	774 (7.8)	

*Includes CS, IS, AS, OS

### Predictors of Escalation and DEXA Scans

To evaluate the likelihood of escalation and the likelihood of obtaining DEXA scans within one year of corticosteroid initiation we conducted logistic regression models adjusting for Veteran-level models factors (age, gender, GI visits) as well as clustering at the facility level. The escalation model included CS and IS patients only (n = 1405 (initially 1678 but excluding those that would have had a single course of corticosteroids before continuous use)) and effect of CS vs. IS. Factors associated with escalation of therapy among the frequent corticosteroid users were age (OR 0.96, 95%CI: 0.95, 0.97), male gender (OR 2.00, 95%CI: 1.16, 3.46), GI visit during steroid evaluation period (OR 8.01,95%CI: 5.85, 10.95) and the use of CS compared to IS (OR 2.28, 95%CI: 1.33,3.90). Factors associated with obtaining a DEXA scans among all patients on corticosteroids (n = 9,881) were male gender (OR 0.55, 95%CI: (0.42, 0.71) and a GI visit prior to corticosteroid initiation (OR 1.38 95%CI: 1.12, 1.70).

## Discussion

In this large cohort of Veteran IBD patients, we found that one-third are treated with corticosteroids at least once during the study period and 15% of the total corticosteroid users were exposed to continuous corticosteroid use. Among these patients with a prolonged exposure, three-quarters did not receive any form of corticosteroid-sparing therapy. The negative effect of corticosteroid exposure was seen in corticosteroid related complications such as VTE, fragility fractures and infections. Additionally, there was a low rate of utilization of DEXA scans according to society guidelines even accounting for bisphosphonate use.

A similar but much smaller study at a tertiary care facility found that 77% of the IBD patients had been on corticosteroids for more than 3 months and almost 60% had not had an attempted escalation of therapy [15]. Additionally, 78% of those on corticosteroids had not undergone DEXA scanning. Our study is a robust representation of national practice patterns in the VHA due to the size of the cohort studied and the fact that our results are not subject to the referral bias present at a tertiary care center. More specifically, it provides additional information regarding patients who are not referred to a tertiary care facility for either expert care or for more complicated disease. This study also provides a unique look at the use of corticosteroids in a national cohort in the modern era. An examination of the corticosteroid use of IBD patients in Olmsted County from the 1970s to 1993 showed that approximately 40% of IBD patients received corticosteroids during that time period [16]. Of these patients, approximately a quarter remained corticosteroid dependent after 1 year but it should be noted that this was prior to the era of anti-TNF therapy. In our population, approximately one-third of Veterans required corticosteroids (CS, IS, AS & OS).

There are several limitations to our study. It is possible that our results may be inaccurate due to an inability to account for care that occurs outside of the VHA. All Veterans are eligible at age 65 for Medicare and VHA care. Studies of patients who have access to both systems reveal that more than 60% utilize specialty care outside of the VHA [17]. Almost 70% of our patients on persistent corticosteroids did not have a recorded GI visit during the period of corticosteroid use. This is most likely attributable to care sought outside of the VHA. While these patients may seek care outside of the system, we postulate that this would only underestimate their corticosteroid use and that many Veterans would continue to obtain prescriptions through the VHA due to cheaper copays for the more expensive medications. In addition, a sensitivity analysis done among Veterans who receive primary care at the VHA (based on at least one primary care visit at the VHA within 365 days after their study period index IBD date) showed similar patterns of results. There may be issues with the generalizability of our findings to IBD care throughout the US due to demographic differences such as age and gender. For comparison in the US overall, the median age of diagnosis of IBD is approximately 30 with a slight male predominance [18], whereas the VHA has an older population of veterans who are predominantly male. The gender difference theoretically should not play a role in the decision to escalate therapy or perform appropriate healthcare maintenance. This cohort’s older average age, however, may have had some influence on the decision to escalate to more aggressive therapy. While providers may be hesitant to escalate therapy, the risk of fragility fractures, VTE, and other complications among the elderly on chronic corticosteroids is not insignificant and carries significant morbidity and mortality [19, 20].

The VHA is the largest healthcare delivery system in the country and is known for delivering high quality care in those who access the system [21, 22]. This high quality care is in large part due to the electronic medical record system which can alert providers to diagnosis-specific quality measures. This electronic medical record system could be harnessed to improve the quality of care among the IBD population much as it has been used to improve the care of patients with diabetes [22]. While it would be easy to make changes to the system to improve the rates of DEXA scanning in this population, improving the initiation of corticosteroid-sparing medications would be more difficult to successfully implement. A more practical algorithm to improving care in the VHA for this particular quality measure would be a stimulus to ensure a recent or future GI visit when corticosteroids are prescribed for patients with an IBD diagnosis with an alert if such a visit is not scheduled.

## Conclusion

Prolonged corticosteroid therapy for the treatment of IBD is common and may be associated with significant harm to patients. Despite this, there was low utilization of preventative measures such as DEXA scans. No individual-level or facility-level factors were specifically associated with use of corticosteroid-sparing medications; however, a gastroenterology visit was associated with an increased likelihood of using corticosteroid-sparing medications. Patients with prolonged use of corticosteroids for IBD should be referred to gastroenterology early and universal efforts to improve the delivery of high quality care should be undertaken.

## Supporting Information

S1 Appendices Appendix A: Generic names of corticosteroids. Appendix B: ICD-9-CM codes for a variety of common inflammatory comorbid conditions. Appendix C: ICD-9-CM codes used to identify VTE, fragility fractures and infections.
(DOCX)

S1 Tables Supplementary Results of patient characteristics and patterns of corticosteroid use. Supplementary Table A: Patient Characteristics (N = 30,456). Supplementary Table B: Patterns of Corticosteroid user characteristics among Veteran with and without corticosteroids for IBD only.
(DOCX)

## Abbreviations

IBD: inflammatory bowel disease
