# Correction: Corticosteroid Use and Complications in a US Inflammatory Bowel Disease Cohort

**DOI:** 10.1371/journal.pone.0197341

**Published:** 2018-05-09

**Authors:** Akbar K. Waljee, Wyndy L. Wiitala, Shail Govani, Ryan Stidham, Sameer Saini, Jason Hou, Linda A. Feagins, Nabeel Khan, Chester B. Good, Sandeep Vijan, Peter D. R. Higgins

In Table 1, the labels “Ulcerative Colitis” and “Crohn’s Disease” are swapped in the first column. Please see the corrected Table 1 here.

**Table 1:** Patient characteristics of no corticosteroid and all corticosteroid users among IBD Veterans.

		Total	No Corticosteroid Users (NS)	All Corticosteroid Users *	P-value
No. of Patients, N (%)		30,456 (100)	20,575 (67.6)	9,881 (32.4)	
Crohn’s Disease, N (%)		10,664 (35.0)	7,126 (34.6)	3,538 (35.8)	0.045
Ulcerative Colitis, N (%)		16,429 (53.9)	11,948 (58.1)	4,481 (45.3)	<0.001
Indeterminate Colitis, N (%)		3,363 (11.0)	1,501 (7.3)	1,862 (18.8)	<0.001
Male, N (%)		28,500 (93.6)	19,389 (94.2)	9,111 (92.2)	<0.001
Age, years Mean (SD)		60.3 (15.3)	62.3 (14.5)	56.2 (16.0)	<0.001
Race, N (%)					<0.001
	Caucasian	21,010 (69.0)	13,898 (67.5)	7,112 (72.0)	
	African American	2,097 (6.9)	1,199 (5.8)	898 (9.1)	
	Other	491 (1.6)	298 (1.4)	193 (2.0)	
	Unknown or Missing	6,858 (22.5)	5,180 (25.2)	1,678 (17.0)	
Any GI Visit, N (%)		15,832 (51.9)	8,332 (40.5)	7,500 (75.9)	<0.001

* Includes CS, IS, AS, OS

In Supplementary Table A of S1 Tables, the labels “UC” and “CD” are swapped in the first column. Please see the correct Supplementary Table A here.

## Supporting information

S1 Tables Supplementary results of patient characteristics and patterns of corticosteroid use. Supplementary Table A: Patient Characteristics (N = 30,456). Supplementary Table B: Patterns of Corticosteroid user characteristics among Veteran with and without corticosteroids for IBD only.
(DOCX)
