# Supplementary material for: Corticosteroid Use and Complications in a US Inflammatory Bowel Disease Cohort
Source: PLoS One. 2016 Jun 23;11(6):e0158017. doi: 10.1371/journal.pone.0158017 (PMC4918923; doi:10.1371/journal.pone.0158017)
Supplement: S1 Appendices — Appendix A: Generic names of corticosteroids. Appendix B: ICD-9-CM codes for a variety of common inflammatory comorbid conditions. Appendix C: ICD-9-CM codes used to identify VTE, fragility fractures and infections. (DOCX) [file pone.0158017.s001.docx]

Appendix A: Generic names of corticosteroids

Types of corticosteroids include prednisone, methylprednisolone, hydrocortisone, cortisone, tixocortol pivalate, prednisolone, budesonide, pivalone, or methylprednisone.

Appendix B: ICD-9-CM codes for a variety of common inflammatory comorbid conditions

Excluded comorbidities based on ICD-9-CM codes within 7 days of a corticosteroid prescription. [491.x, 492.x, 496.x for chronic obstructive pulmonary disease, 493.x for asthma, 714.x for rheumatoid arthritis, 710.x for lupus, 69x.x for dermatitis, 490.x for bronchitis, 461.x and 473.x for sinusitis, 472.x and 477.x for rhinitis, 725.x for polymyalgia rheumatica, 339.x for cluster headaches, 571.42 and for autoimmune hepatitis, 339.x for cluster headaches occurring within 7 days of a fill were used to identify corticosteroid fills for non-IBD/other conditions] (OS).

Appendix C: ICD-9-CM codes used to identify VTE, fragility fractures and infections

VTE/PE ICD-9-CM codes include: deep vessels (451.1 ×, 451.81, 451.83), vena cava (453.2 ×), lower extremity (453.4 ×), deep phlebothrombosis (671.3 ×,671.4) , pulmonary embolism and infarction (415.1x), iliac vein(451.81) .

Fragility fractures ICD-9-CM codes included: pathological fractures (733.1 ×), lumbar fractures (805.4 ×, 805.5 ×, 806.4 ,×, 806.5×), pelvic fractures (808. ××), distal ulna, and radius fractures (813.4 ×, 813.5 ×) and femoral neck fractures (820. ××).

Infections ICD-9-CM codes include: C. difficile colitis (008.45), Tuberculosis (010.0-018.9)~~,~~ Plague (020. Xx), Tularemia (021. xx ), Anthrax (022. xx ), Brucellosis (023. xx ), Glanders (024. xx ), Melioidosis (025. xx ), Rat-bite fever (026. xx ), Other zoonotic bacterial diseases (027. xx ), Leprosy (030. xx ), Diseases due to other mycobacteria (031. xx ), Diphtheria (032. xx ), Whooping cough (033. xx ), Streptococcal sore throat and scarlet fever (034. xx ), Erysipelas (035. xx ), Meningococcal infection (036. xx ), Tetanus (037. xx ), Septicemia (038. xx ), Actinomycotic infections (039. xx ), Other bacterial diseases (040. xx ), Rickettsia and other arthropod borne diseases, Syphilis and Spirochetes (081.0-104.9), Candidal Infections (112.4-112.89), Blastomycosis, Histoplasmosis, Coccidioidiomycosis and Aspergillosis (114.0-117.9), Toxoplasmosis (130.0-130.9), PCP Pneumonia (136.3), Bacterial meningitis (320. xx ), Intracranial and intraspinal abscess (324. xx ), Orbital cellulitis (376.01), Acute and subacute endocarditis (421. xx ), Peritonsillar abscess (475. xx ), Pneumococcal pneumonia [Streptococcus pneumoniae pneumonia] (481. xx ), Other bacterial pneumonia (482. xx ), Mycoplasma pneumonia (483.0), Chlamydia (483.1), Empyema (510. xx ), Abscess of lung and mediastinum (513. xx ), Diverticulitis of colon without mention of hemorrhage (562.11), Abscess of anal and rectal regions (566. xx ), Peritonitis in infectious diseases classified elsewhere (567.0), Pneumococcal peritonitis (567.1), Other suppurative peritonitis (567.2), Retroperitoneal infections (567.3), Abscess of liver (572.0), Acute cholecystitis (575. 0 ), Cholangitis (576.1), Acute pyelonephritis (590.1), Renal and perinephric abscess (590.2), Abscess of prostate (601.2), Arthropathy associated with infections (711. xx ), Necrotizing fasciitis (728.86), Osteomyelitis, periostitis, and other infections involving bone (730. xx ), Septic shock (785.52), Sepsis (995.91), Severe sepsis (995.92).
