# Supplementary material for: Corticosteroid Use and Complications in a US Inflammatory Bowel Disease Cohort
Source: PLoS One. 2016 Jun 23;11(6):e0158017. doi: 10.1371/journal.pone.0158017 (PMC4918923; doi:10.1371/journal.pone.0158017)
Supplement: S1 Tables — Supplementary Table A: Patient Characteristics (N = 30,456). Supplementary Table B: Patterns of Corticosteroid user characteristics among Veteran with and without corticosteroids for IBD only. (DOCX) [file pone.0158017.s002.docx]

**Supplementary Results:**

*Patterns of Corticosteroid User*

14.6% of Veterans on corticosteroids required continuous corticosteroids use (CS) based on our definition, 2.4% required IS, 44.3% AS and 38.7% OS. Supplementary Tables A and B provide the Veteran demographics and a detailed breakdown of the patterns of corticosteroid use for inflammatory bowel disease. The IS and AS groups were younger, on average (52±17 and 52±16, respectively), than the NS group (62±15), CS group (58±16), and OS group (61±14), p<0.001. Corticosteroid users were more likely to have a GI visit during the study period (71.8% of CS; 80.4% of IS; 83.3% of AS), compared to NS users (40.5% of NS), p<0.001. Furthermore, there were significant differences in whether or not patients in the corticosteroid groups had a gastroenterology (GI) visit within 365 days of their corticosteroid initiation date: 57.7% of CS users had a GI visit after their corticosteroid initiation, compared to 70% of AS users and 67.5% of IS users, p<0.001. Gastroenterology visits had a significant effect on the use of corticosteroids and the proportion of study days spent on corticosteroids in the CS, IS, and AS groups within 365 days. CS users who had a GI visit had a lower proportion of days on corticosteroids (median=0.14, 5^th^ – 95^th^ percentile: 0.03 – 0.74) compared to those who did not have a GI visit (median=0.20, 5^th^ – 95^th^ percentile: 0.03 – 0.90), p<0.001. IS users with a GI visit had similar proportion of days on corticosteroids (median=0.06, 5^th^ – 95^th^ percentile: 0.01 – 0.26) compared to those who did not have a GI visit (median=0.05, 5^th^ – 95^th^ percentile: 0.01 – 0.28), p =0.654. AS users with a GI visit, on the other hand, had greater proportion of study days on corticosteroids (median=0.05, 5^th^ – 95^th^ percentile: 0.007 – 0.37) than those who did not have a GI visit (median=0.03, 5^th^ – 95^th^ percentile: 0.004 – 0.26), p<0.001.

Supplementary Table A: Patient Characteristics (N=30,456)

|  | **Total** | **Non corticosteroids**  **Users (NS)** | **Continuous**  **corticosteroid Users (CS)** | **Intermittent corticosteroid**  **Users (IS)** | **Any**  **corticosteroid**  **Users (AS)** | **Other**  **corticosteroid**  **Users (OS)** | **p** |
| --- | --- | --- | --- | --- | --- | --- | --- |
| No. of Patients | 30,456 (100) | 20,575  (67.6) | 1,438  (4.7) | 240  (0.8) | 4,376  (14.4) | 3,827  (12.6) |  |
| UC | 10,664  (35.0) | 7,126  (34.6) | 512  (35.6) | 61  (25.4) | 1,610  (36.8) | 1,355  (35.4) | 0.001 |
| CD | 16,429  (53.9) | 11,948  (58.1) | 631  (43.9) | 131 (54.6) | 1,809  (41.3) | 1,910  (49.9) | <0.001 |
| IC | 3,363  (11.0) | 1,501  (7.3) | 295  (20.5) | 48  (20.0) | 957  (21.9) | 562  (14.7) | <0.001 |
| Male, n (%) | 28,500 (93.6) | 19,389 (94.2) | 1,356  (94.3) | 218  (90.8) | 4,022  (91.9) | 3,515  (91.9) | <0.001 |
| Age, (SD) | 60  ± 15 | 62  ± 15^a^ | 58  ± 16^b^ | 52  ± 17^c^ | 52  ± 16^c^ | 61  ± 14^d^ | <0.001 |
| Race, n (%) |  |  |  |  |  |  | <0.001 |
| Caucasian | 21,010 (69.0) | 13,898 (67.6) | 1,018  (70.8) | 168  (70.0) | 3,157  (72.1) | 2,769  (72.4) |  |
| African American | 2,097 (6.9) | 1,199  (5.8) | 122  (8.5) | 31  (12.9) | 406  (9.3) | 339  (8.9) |  |
| Other | 491  (1.6) | 298  (1.4) | 25  (1.7) | 7  (2.9) | 93  (2.1) | 68  (1.8) |  |
| Unknown or Missing | 6,858 (22.5) | 5,180 (25.2) | 273  (19) | 34  (14.2) | 720  (16.5) | 651  (17.0) |  |

Note. Frequency and percent are displayed for categorical measures and mean ± standard deviation is displayed for continuous measures. Means with different superscripts are significantly different, p<0.05, based on one-way ANOVA with Scheffe post-hoc tests.

Supplementary Table B: Patterns of Corticosteroid user characteristics among Veteran with and without corticosteroids for IBD only.

|  | **Non corticosteroids**  **Users (NS)** | **Continuous**  **corticosteroid Users (CS)** | **Intermittent corticosteroid**  **Users (IS)** | **Any**  **corticosteroid**  **Users (AS)** | **p** |
| --- | --- | --- | --- | --- | --- |
| No. of Patients | 20,575  (77.3) | 1,438  (5.4) | 240  (0.9) | 4,376  (16.4) |  |
| Study Days  (5-95 percentile) | 2,032  (508 – 3,173)^a^ | 2,236  (639 – 3,200)^b^ | 2,180  (525 – 3,173)^b^ | 2,051  (528 – 3,179)^a^ | <0.001 |
| Rate of  Escalation, n (%) | 1,210  (5.9) | 384  (26.7) | 55  (22.9) | 2,089  (47.7) | <0.001 |
| Type of Escalation Medication, (n=1,210) |  |  |  |  |  |
| Anti-TNF only | 85  (7.0) | 49  (12.8) | 7  (12.7) | 490  (23.5) |  |
| Immuno only | 1,064  (87.9) | 298  (77.6) | 46  (83.6) | 1,319  (63.1) |  |
| Combination | 61  (5.0) | 37  (9.6) | 2  (3.6) | 280  (13.4) |  |
| Any GI visit, n (%) | 8,332  (40.5) | 1,032  (71.8) | 193  (80.4) | 3,644  (83.3) | <0.001 |

* % Exclude the OS group

Note. Medians with different superscripts are significantly different, based on Kruskal-Wallis test using Dunn Test post-hoc comparisons, p<0.05.
